# Transcriptional Network Analysis Reveals that AT1 and AT2 Angiotensin II Receptors Are Both Involved in the Regulation of Genes Essential for Glioma Progression

**DOI:** 10.1371/journal.pone.0110934

**Published:** 2014-11-03

**Authors:** Hátylas Azevedo, André Fujita, Silvia Yumi Bando, Priscila Iamashita, Carlos Alberto Moreira-Filho

**Affiliations:** 1 Department of Pediatrics, Faculdade de Medicina da Universidade de São Paulo (FMUSP), São Paulo, SP, Brazil; 2 Department of Computer Science, Instituto de Matemática e Estatística, Universidade de São Paulo, São Paulo, SP, Brazil; Cleveland Clinic Lerner Research Institute, United States of America

## Abstract

Gliomas are aggressive primary brain tumors with high infiltrative potential. The expression of Angiotensin II (Ang II) receptors has been associated with poor prognosis in human astrocytomas, the most common type of glioma. In this study, we investigated the role of Angiotensin II in glioma malignancy through transcriptional profiling and network analysis of cultured C6 rat glioma cells exposed to Ang II and to inhibitors of its membrane receptor subtypes. C6 cells were treated with Ang II and specific antagonists of AT1 and AT2 receptors. Total RNA was isolated after three and six hours of Ang II treatment and analyzed by oligonucleotide microarray technology. Gene expression data was evaluated through transcriptional network modeling to identify how differentially expressed (DE) genes are connected to each other. Moreover, other genes co-expressing with the DE genes were considered in these analyses in order to support the identification of enriched functions and pathways. A hub-based network analysis showed that the most connected nodes in Ang II-related networks exert functions associated with cell proliferation, migration and invasion, key aspects for glioma progression. The subsequent functional enrichment analysis of these central genes highlighted their participation in signaling pathways that are frequently deregulated in gliomas such as ErbB, MAPK and p53. Noteworthy, either AT1 or AT2 inhibitions were able to down-regulate different sets of hub genes involved in protumoral functions, suggesting that both Ang II receptors could be therapeutic targets for intervention in glioma. Taken together, our results point out multiple actions of Ang II in glioma pathogenesis and reveal the participation of both Ang II receptors in the regulation of genes relevant for glioma progression. This study is the first one to provide systems-level molecular data for better understanding the protumoral effects of Ang II in the proliferative and infiltrative behavior of gliomas.

## Background

Gliomas are highly prevalent and therapy-resistant types of primary brain cancer. Despite recent advances in glioma therapy, the current standard therapeutic procedure still comprises maximum surgical resection and radiotherapy with temozolomide [1]. Patients undergoing this procedure have a median survival time of less than 2 years, illustrating how the prognosis of glioma patients is bleak. Surgical treatment presents many limitations, as the infiltrative nature of these tumors causes them to diffuse around surrounding brain parenchyma [2]. Consequently, molecular mechanisms underlying the poor prognosis of patients with gliomas should be investigated in order to develop novel drug-based treatments for blocking tumor progression. An interesting clue for unraveling those mechanisms is given by the association between expression of Angiotensin II (Ang II) receptors and poor prognosis in human astrocytomas [3].

The peptide Ang II is the main effector of the renin-angiotensin system and exerts its effects by the activation of two selective receptor subtypes named AT1 and AT2 [4]. Ang II was firstly described as a key regulatory factor in blood pressure control. However, non-canonical functions of Ang II such as cell proliferation, apoptosis and angiogenesis were recently described in malignant neoplasms [5]–[8]. Targeting Ang II signaling may impede tumor progression in patients and experimental models of cancer [9]–[11], as the invasiveness and immunosuppression state of many types of cancer is dependent on the up-regulation of AT1 receptor [12], [13]. Consequently, AT1 has been established as a potential therapeutic target in cancer. On the other hand, the role of AT2 in neoplasias is poorly investigated and remains controversial. While some authors state that AT2 is mostly associated with protumoral functions [14], [15], others indicate that it is involved in carcinogenesis [16].

Different glioma cell lines express AT1 and AT2 receptors and show a mitogenic response when incubated with Angiotensin peptides [17]. Indeed, blocking AT1 receptor decreases the synthesis of growth factors, induces apoptosis and reduces the growth of cultured C6 glioma cells and C6 rat glioma *in vivo* [18], [19]. However, the molecular mechanisms underlying the protumoral functions of Ang II are not fully described. Given the known role of Ang II in transcriptional regulation [20]–[23], it is worth investigating Ang II effects on glioma cells focusing on transcriptional profile changes and the corresponding modifications in gene interaction networks.

Oligonucleotide microarray profiling is a powerful tool for disclosing gene expression patterns associated with cell events [24]. This profiling coupled with bioinformatics analysis enables the identification of biological functions downstream of receptor activation, as well as how the differentially expressed genes behave in transcriptional networks. To date, there is a lack of information on network modeling of Ang II transcriptional effects in glioma cells. In this way, we sought here to reveal the transcriptional networks modulated by Ang II in C6 glioma cells via AT1 and AT2 receptors. Our results contribute to unravel the molecular program initiated by the activation of Ang II receptors in C6 cells, shedding a light on Ang II roles in glioma progression.

## Results

### Comparative transcriptomic analysis of Ang II effects on C6 glioma cells

We carried out microarray experiments to find gene expression changes associated with Ang II treatment in C6 glioma cells. The treatment scheme (see Methods) was designed to address the individual contribution of AT1 and AT2 receptors in the transcriptomic changes mediated by Ang II, using specific antagonists of AT1 and AT2 – respectively Losartan and PD123319 [25] - in separate groups. The time intervals of 3 and 6 hours were selected following a previous study showing a slight but significant increase in C6 cells proliferation after 6 hours treatment with Ang II [18]. Taking this into consideration, we were specifically interested in evaluating transcriptional events preceding an increase in cell proliferation. Moreover, early but not chronic transcriptional changes are more likely to be directly induced by Ang II treatment. In parallel, human adrenocarcinoma cells stimulated with Ang II had maximum expression levels for all genes occurring 3 to 6 h after Ang II stimulation [26]. Therefore, it is reasonable to consider that relevant changes in the transcriptomic profile of C6 cells may occur within the first 6 hours of Ang II exposure in an *in vitro* setting.

Differentially expressed (DE) genes in each comparison were identified using t-tests with p<0.05. Most of the DE genes had their expression only slightly changed at the time intervals studied here, ranging from 1.2 to 3 fold changes. The statistical comparison between the Ang II-treated and Control groups disclosed which genes had their expression levels changed due to the activation of both AT1 and AT2 receptors by Ang II. On the other hand, the statistical comparison between the group treated with Ang II plus Losartan and that treated only with Ang II revealed DE genes regulated by AT1 receptor. Analogously, the statistical comparison between Ang II plus PD123319 and Ang II only-treated groups disclosed which DE genes were regulated by AT2 receptor. Tables S1 to S12 in File S1 list the DE genes that appeared in the functional enrichment analysis, according to Gene Ontology (GO) and KEGG databases. Table S13 in File S2 and Table S14 in File S3 lists the DE gene's p-value and fold changes for all comparisons at 3 and 6 hours, respectively.

### Identification of commonly regulated genes across the comparisons

**Figure 1:**
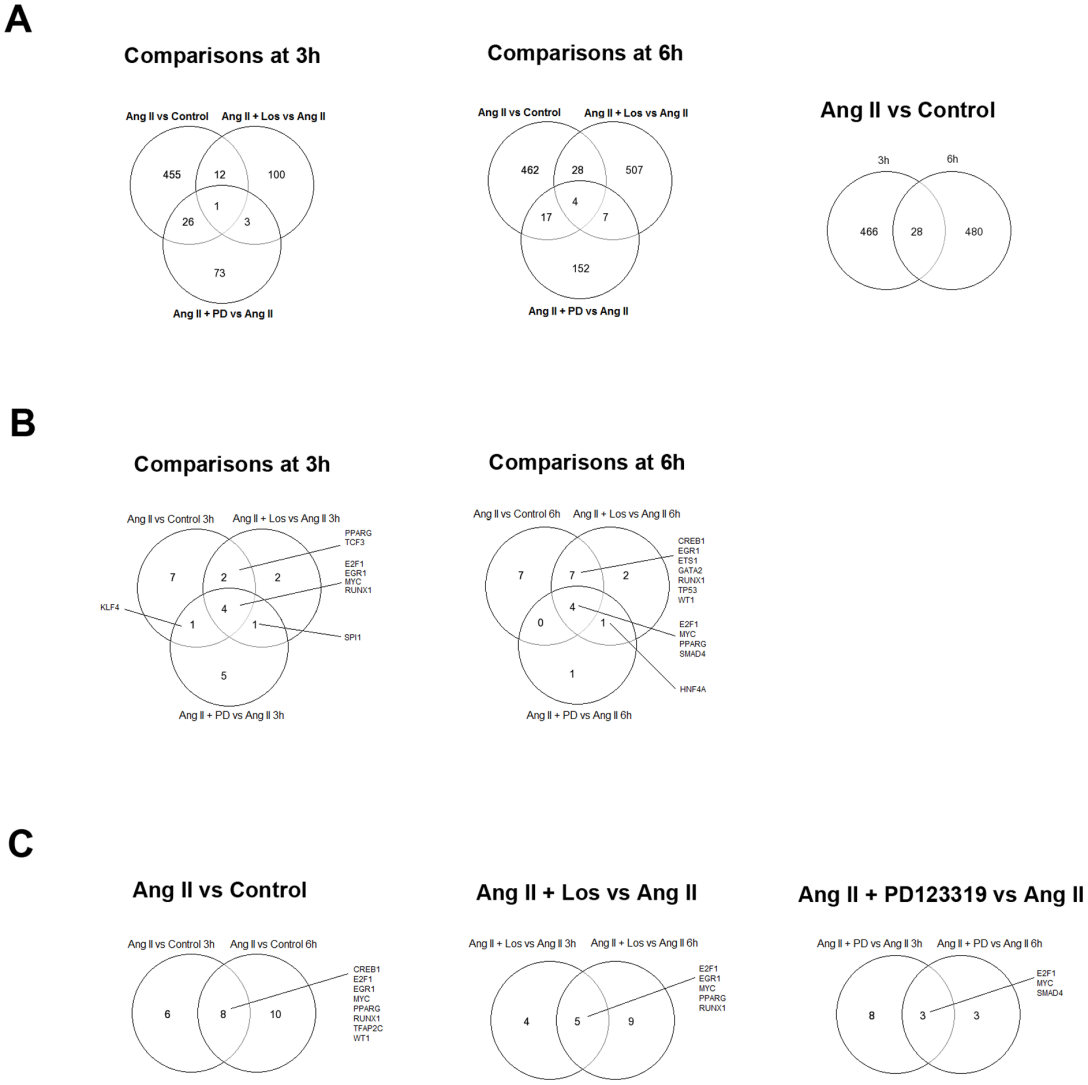
Venn diagrams showing the number of common DE genes and the common enriched transcription factors across the experimental comparisons. (A) Number of common and exclusively regulated genes at 3 and 6 hours intervals for Ang x Control, Ang II+Los x Ang II and Ang II+PD123319 x Ang II comparisons. (B) Number and enriched transcription factors observed when each time interval was analyzed separately. (C) Number and enriched transcription factors observed for the same comparisons at both 3 and 6 hours time intervals.

Venn diagrams were constructed using DE genes obtained in all experimental comparisons in order to identify: i) DE genes regulated by Ang II at both 3 and 6 hours intervals, or ii) genes whose expression is altered by Ang II and by the presence of Ang II and its antagonists. Genes encompassed in these overlaps are thereafter called common genes (Figure 1A and Table S15 in File S4). Interestingly, Ang II x Control comparisons presented the largest set of common genes (28 DE genes). From these genes, the gene Rev3L, encoding the catalytic subunit of human polymerase zeta, was previously associated with temozolomide resistance and DNA repair mechanisms [27]. Another gene encoding the protein Zyxin (Zyx) was also regulated at both time intervals. Zyx concentrates at focal adhesions, regulates actin assembly and was previously described to be relevant for cell migration and invasion [28]. In addition, the Nek2 gene, which codes for a centrosomal kinase, was also observed in the common genes list obtained from Ang II x Control comparisons. Nek2 overexpression was shown to confer an inferior survival in gliomas and it is associated with drug resistance and cell proliferation in several types of cancer [29]. Finally, the Pias1 gene, which encodes an inhibitor of signal transducer and activator of transcription1 (STAT-1), was also found in this gene set. PIAS family members were already showed to be greatly reduced in glioblastomas, resulting in overactivation of STAT-dependent transcription [30].

### Enrichment of transcription factors across differential gene expression profiles

Enriched transcription factors (TFs) were identified from the differential datasets in order to reveal upstream regulatory pathways modulating gene expression changes. With this approach, we were able to identify transcription factors that are downstream of AT1R and AT2R activation in glioma cells. We constrained our further analysis on the 100 top statistically enriched TFs displayed in both ChEA and Transfac databases. The set lists of enriched TFs are depicted in Table S16 in File S5. Then, the enriched TFs were used for building Venn diagrams in order to identify TFs regulated by Ang II at both 3 and 6 hours intervals and those modulated by Ang II but also by the presence of Ang II and its antagonists (Figure 1B and 1C). This analysis disclosed TFs potentially associated with the upstream regulation of DE genes by Ang II in each comparison, such as Creb1, E2f1, Egr1, Myc, Runx1, Tfap2c, Ets1 and Wt1. In fact, the transcription factors Creb1, E2f1, Egr1, Myc and Ets1 were described to be regulated by Ang II in previous studies [31]–[35], confirming our *in silico* results.

### Transcriptional network analysis reveals biological functions and hierarchical characteristics of genes regulated by Ang II

Gene interaction networks were generated to shed light on the patterns of gene-gene interactions from the measured datasets of gene expression. We included in the networks the DE genes obtained for each comparison and the genes that co-express with the respective DE genes (DE-related genes). DE-related genes were found through the Cytoscape plug-in GeneMANIA and included in the network analysis to verify how DE genes interact with other co-expressed genes according to data computed in GeneMANIA. The resulting networks containing DE and DE-related genes allowed the search for functions and pathways related to Ang II role in glioma progression, as described below.

In a network model, genes are represented as nodes in a graphic visualization, while functional relationships (e.g. protein-protein interactions, transcription regulation, gene co-expression, etc.) are represented as edges connecting the corresponding nodes. The information about functional relationships is usually obtained through different databases [36]. Moreover, the mathematical analysis of the connections between nodes is usually applied to reveal emergent properties of these networks [37].

**Figure 2:**
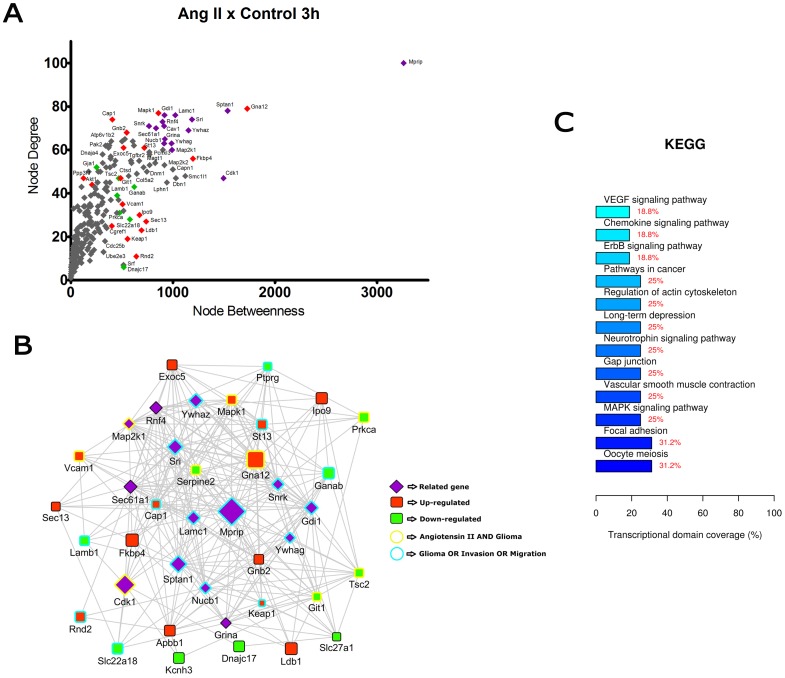
Transcriptional network enrichment analysis of hub genes found at Ang II x Control 3 h comparison. (A) Scatter plot of betweenness centrality versus degree for nodes obtained in the transcriptional network analysis. Differentially expressed (DE) genes are represented as red (up-regulated) or green (down-regulated) diamonds in the graphic. DE-related genes are represented as purple diamonds. (B) Transcriptional interaction subnetwork containing the 25 DE genes and 15 DE-related genes with the highest centrality values in each network. DE genes are represented as red (up-regulated) or green (down-regulated) squares in the networks. DE-related genes are represented as purple diamonds. Genes previously associated with the keywords “Angiotensin II” and “glioma” display yellow border colors. Genes previously associated with the keywords “glioma”, “migration” or “invasion” display sea green border colors. (C) KEGG categories showing enrichment in functions for the hub genes.

**Figure 3:**
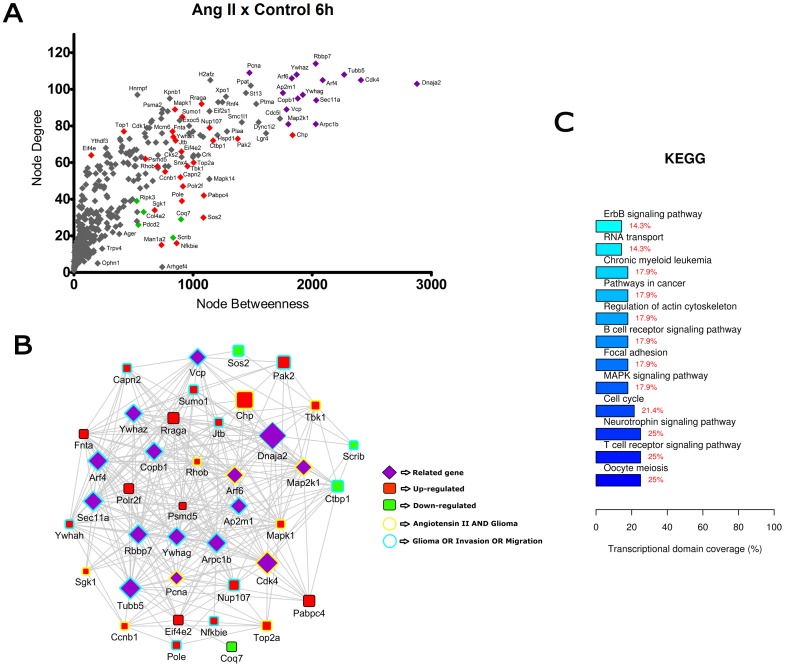
Transcriptional network enrichment analysis of hub genes found at Ang II x Control 6 h comparison. (A) Scatter plot of betweenness centrality versus degree for nodes obtained in the transcriptional network analysis. Differentially expressed (DE) genes are represented as red (up-regulated) or green (down-regulated) diamonds in the graphic. DE-related genes are represented as purple diamonds. (B) Transcriptional interaction subnetwork containing the 25 DE genes and 15 DE-related genes with the highest centrality values in each network. DE genes are represented as red (up-regulated) or green (down-regulated) squares in the networks. DE-related genes are represented as purple diamonds. Genes previously associated with the keywords “Angiotensin II” and “glioma” display yellow border colors. Genes previously associated with the keywords “glioma”, “migration” or “invasion” display sea green border colors. (C) KEGG categories showing enrichment in functions for the hub genes.

**Figure 4:**
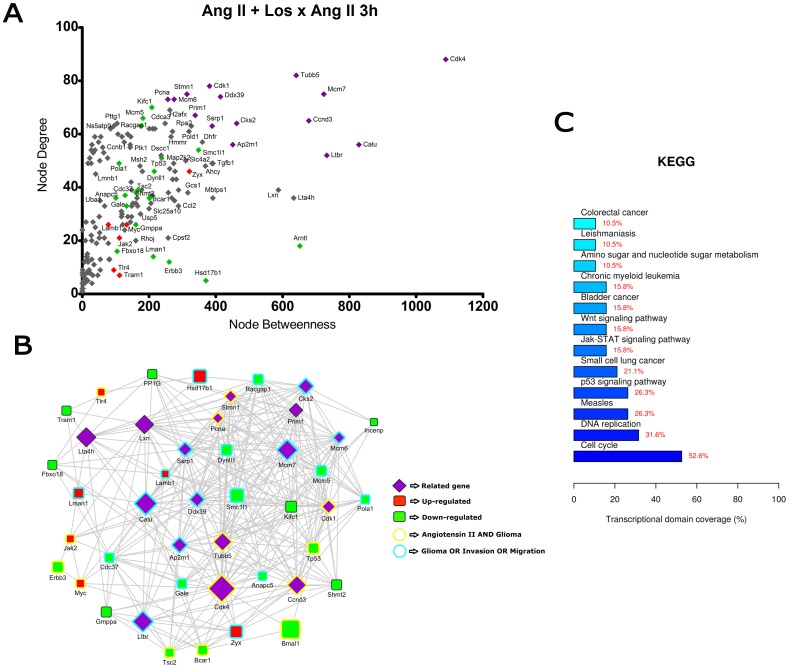
Transcriptional network enrichment analysis of hub genes found at Ang II + Losartan x Ang II 3 h comparison. (A) Scatter plot of betweenness centrality versus degree for nodes obtained in the transcriptional network analysis. Differentially expressed (DE) genes are represented as red (up-regulated) or green (down-regulated) diamonds in the graphic. DE-related genes are represented as purple diamonds. (B) Transcriptional interaction subnetwork containing the 25 DE genes and 15 DE-related genes with the highest centrality values in each network. DE genes are represented as red (up-regulated) or green (down-regulated) squares in the networks. DE-related genes are represented as purple diamonds. Genes previously associated with the keywords “Angiotensin II” and “glioma” display yellow border colors. Genes previously associated with the keywords “glioma”, “migration” or “invasion” display sea green border colors. (C) KEGG categories showing enrichment in functions for the hub genes.

**Figure 5:**
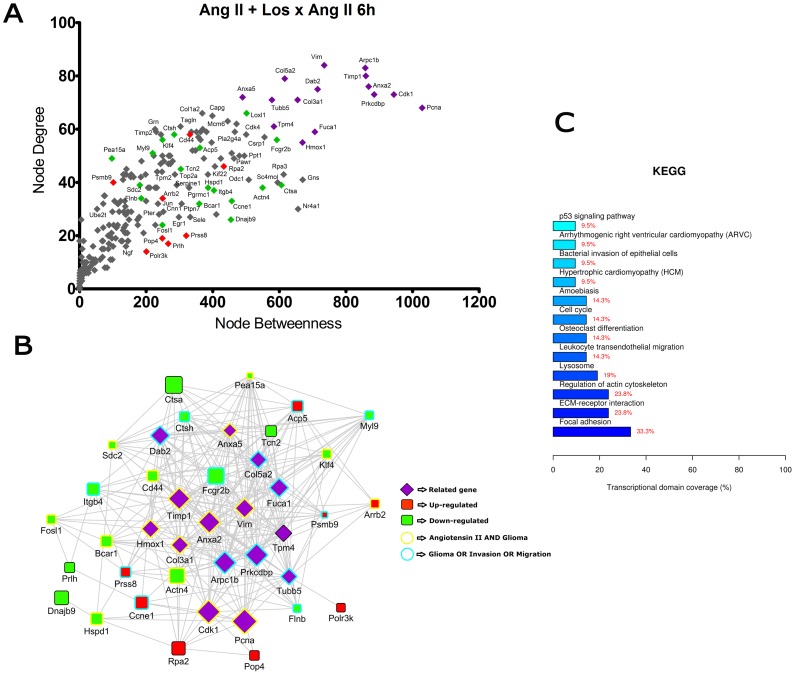
Transcriptional network enrichment analysis of hub genes found at Ang II + Losartan x Ang II 6 h comparison. (A) Scatter plot of betweenness centrality versus degree for nodes obtained in the transcriptional network analysis. Differentially expressed (DE) genes are represented as red (up-regulated) or green (down-regulated) diamonds in the graphic. DE-related genes are represented as purple diamonds. (B) Transcriptional interaction subnetwork containing the 25 DE genes and 15 DE-related genes with the highest centrality values in each network. DE genes are represented as red (up-regulated) or green (down-regulated) squares in the networks. DE-related genes are represented as purple diamonds. Genes previously associated with the keywords “Angiotensin II” and “glioma” display yellow border colors. Genes previously associated with the keywords “glioma”, “migration” or “invasion” display sea green border colors. (C) KEGG categories showing enrichment in functions for the hub genes.

**Figure 6:**
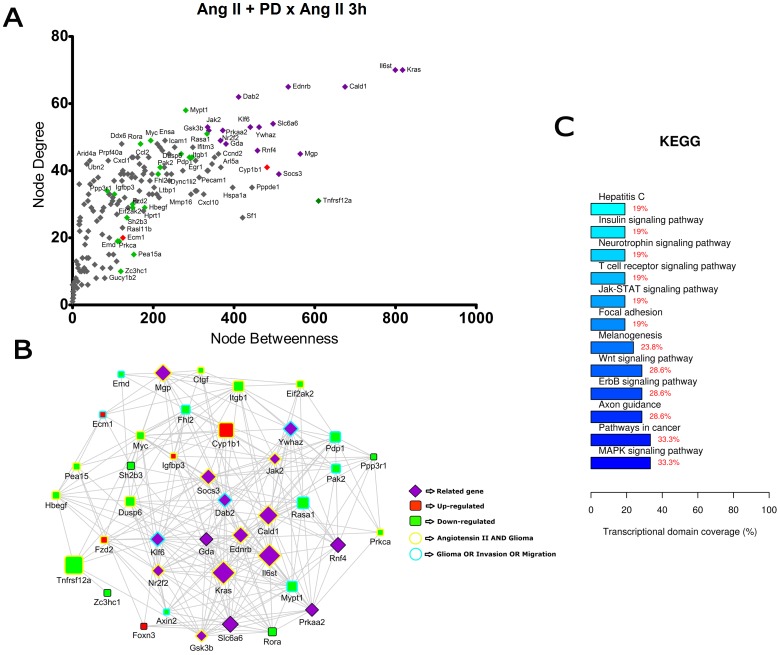
Transcriptional network enrichment analysis of hub genes found at Ang II +PD123319 x Ang II 3 h comparison. (A) Scatter plot of betweenness centrality versus degree for nodes obtained in the transcriptional network analysis. Differentially expressed (DE) genes are represented as red (up-regulated) or green (down-regulated) diamonds in the graphic. DE-related genes are represented as purple diamonds. (B) Transcriptional interaction subnetwork containing the 25 DE genes and 15 DE-related genes with the highest centrality values in each network. DE genes are represented as red (up-regulated) or green (down-regulated) squares in the networks. DE-related genes are represented as purple diamonds. Genes previously associated with the keywords “Angiotensin II” and “glioma” display yellow border colors. Genes previously associated with the keywords “glioma”, “migration” or “invasion” display sea green border colors. (C) KEGG categories showing enrichment in functions for the hub genes.

**Figure 7:**
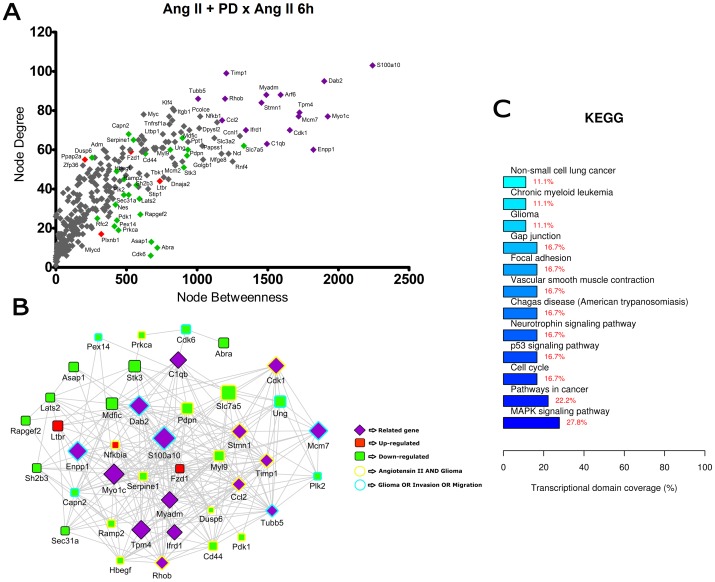
Transcriptional network enrichment analysis of hub genes found at Ang II +PD123319 x Ang II 6 h comparison. (A) Scatter plot of betweenness centrality versus degree for nodes obtained in the transcriptional network analysis. Differentially expressed (DE) genes are represented as red (up-regulated) or green (down-regulated) diamonds in the graphic. DE-related genes are represented as purple diamonds. (B) Transcriptional interaction subnetwork containing the 25 DE genes and 15 DE-related genes with the highest centrality values in each network. DE genes are represented as red (up-regulated) or green (down-regulated) squares in the networks. DE-related genes are represented as purple diamonds. Genes previously associated with the keywords “Angiotensin II” and “glioma” display yellow border colors. Genes previously associated with the keywords “glioma”, “migration” or “invasion” display sea green border colors. (C) KEGG categories showing enrichment in functions for the hub genes.

Centrality measures (degree and betweenness) were used here to investigate the topological characteristics of the nodes in the networks. While node degree identifies the number of connections incident upon a node, node betweenness discriminates the relative central position of a certain node in the network by calculating the number of shortest paths passing through a specific node. These centrality measures allowed the detection of highly connected and central genes - the hubs - in each network. We next built scatter plots showing the relationship among betweenness and degree values for each node in order to identify the hub genes within the networks. These scatter plots allowed the selection of 25 DE genes and 15 co-expressed genes with the highest centrality values in each network. These top-ranked genes were used to build network graphic representations and were further analyzed to identify enriched biological functions, as depicted in Figures 2 to 7.

In the comparisons Ang II versus Control at 3 h and 6 h (Figures 2 and 3), most of the hubs were associated with focal adhesion, regulation of actin cytoskeleton, cell cycle and signaling pathways ErbB, VEGF and MAPK. Interestingly, anaphase-promoting complex genes (Anapc10, Cdc20, Cdc26, Psmd14, Psma1, Bub1b, Nup107 and Ccnb1) were differentially expressed in the Ang II-treated group. In the comparisons Ang II plus Losartan versus Ang II at 3 h and 6 h, many hubs were related to focal adhesion, regulation of actin cytoskeleton, cell cycle, ECM-receptor interaction as well as to MAPK, p53 and Wnt signaling pathways. On the other hand, in Ang II plus PD123319 versus Ang II comparisons at 3 h and 6 h, hubs were associated with pathways in cancer, focal adhesion and signaling pathways ErbB, Wnt, p53, neurotrophin and MAPK. Of interest, treatment with either Losartan or PD123319 mostly down-regulated the expression of genes associated with the protumoral processes described above. This can be clearly observed in the comparisons Ang II plus Losartan versus Ang II (Figures 4 and 5) or Ang II plus PD123319 versus Ang II (Figures 6 and 7), where the subnetworks obtained for genes with highest centrality contain a larger number of nodes corresponding to down-regulated genes.

Text mining using GenClip revealed that most of these hubs have been associated with the keywords “Ang II” and “gliomas” or with the keywords “glioma”, “migration” and “invasion”. These hubs are represented in the networks with yellow and sea green border colors, respectively. Finally, we selected for each of the transcription interaction networks the top five DE and DE-related genes, according to their node betweenness values. Main literature findings were highlighted for these genes to provide further information on their role on tumor progression (Tables 1 and 2).

**Table 1:** List of top-ranked hubs identified in the transcriptional networks derived from Ang II vs Control 3 h, Ang II vs Control 6 h and Ang II + Los vs Ang II 3 h comparisons.

Comparison	Type	Gene ID	Betweenness	Degree	Gene	Tumor Relevance	Ref.
**Ang x Control 3 h**	related	116504	3260,2	100	Mprip	target of JNK signaling, required for cancer cell invasion	[83]
	related	64159	1535,9	78	Sptan1	associated with chemoresistant ovarian tumors	[84]
	related	117036	1022,8	76	Lamc1	up-regulated in gliomas and involved in migration and invasion	[85]
	related	25183	917,1	76	Gdi1	RhoA signaling and actin cytoskeleton in glioma cells	[86]
	related	683667	1187,1	74	Sri	histological markers for glioblastoma multiforme	[87]
	DE	81663	1727,8	79	Gna12	G-protein signaling component associated with glioma cell motility	[88]
	DE	260321	1196,2	56	Fkbp4	promotes assembly of the Hsp90 chaperone complex and stimulates cell growth	[89]
	DE	116590	857,7	77	Mapk1	MAPK signaling is activated in pilocytic astrocytoma	[90]
	DE	64185	406,3	74	Cap1	overexpressed in pancreatic tumors and involved in cancer cell motility	[91]
	DE	81667	547,7	68	Gnb2	link integrin engagement with focal adhesion disassembly and cell motility	[92]
**Ang x Control 6 h**	related	83712	2029,2	114	Rbbp7	member of the polycomb group expressed in gliomas, important for self-renewal	[93]
	related	25737	1473,1	109	Pcna	cell cycle biomarker useful to evaluate the proliferating activity of brain tumors	[94]
	related	29214	2268,3	108	Tubb5	associated with cell cycle of progenitors and position of migrating neurons	[95]
	related	25578	1869,4	108	Ywhaz	14-3-3 protein, positive expression associated with poor prognosis in glioblastoma	[96]
	related	79121	1828,3	106	Arf6	Regulates glioma cell invasion	[97]
	DE	117044	1071,5	92	Rraga	relay amino acid signals to TORC1, a central cell growth regulator	[98]
	DE	116590	848,5	89	Mapk1	promising molecular target in glioblastomas	[99]
	DE	301442	910,1	85	Sumo1	activated in human astrocytomas and required for glioblastoma cell survival	[100]
	DE	116555	1138,2	79	Nup107	genomic amplification and overexpression in glioblastoma multiforme	[101]
	DE	25318	827,8	77	Fnta	modyfies Ras with a farnesyl group, its inhibition is particularly relevant in glioblastoma	[102]
**Ang + Los x Ang 3 h**	related	94201	1088,8	88	Cdk4	aberrant expression in human gliomas, its inhibition sensitizes glioma cells	[103]
	related	29214	640,9	82	Tubb5	associated with cell cycle of progenitors and position of migrating neurons	[95]
	related	54237	381,2	78	Cdk1	downregulation can inhibit the proliferation of human gliomas	[104]
	related	288532	723,6	75	Mcm7	stronger increase of MCM7 labelling index in relation to tumor aggressiveness	[105]
	related	303471	313,6	75	Stmn1	candidate gene influencing sensitivity and resistance of glioblastomas to semustine	[106]
	DE	294286	208,4	70	Kifc1	overexpression of kinesins mediates docetaxel resistance in breast cancer cells	[107]
	DE	291885	181,7	66	Mcm5	member of a gene signature for invasive colorectal tumor cells	[108]
	DE	315298	176,9	63	Racgap1	Higher levels of RACGAP1 mRNA were significantly correlated with meningioma progression	[109]
	DE	63996	348,5	54	Smc1l1	knocking down SMC1A inhibits growth and leads to G2/M arrest in human glioma cells	[110]
	DE	24842	237,7	51	Tp53	p53 abnormalities affect the invasive and aggressive nature of malignant astrocytomas	[111]

Top-ranked hubs were identified according to their betweenness centrality values and their relevance in tumor biology was determined. The main literature findings on cancer were highlighted for the top five differentially expressed (DE) genes and DE-related genes. Genes that co-express with DE genes (DE-related genes) were included in the networks to verify how DE genes interact with other functionally related genes.

**Table 2:** List of top-ranked hubs identified in the transcriptional networks derived from Ang II v+ Los vs Ang 6 h, Ang II + PD123319 vs Ang II 3 h and Ang II + PD123319 vs Ang II 6 h comparisons.

Comparison	Type	Gene ID	Betweenness	Degree	Gene	Tumor Relevance	Ref.
**Ang + Los x Ang 6 h**	related	25737	1028,8	68	Pcna	cell cycle biomarker useful to evaluate the proliferating activity of brain tumors	[94]
	related	54237	943,9	73	Cdk1	downregulation can inhibit the proliferation of human gliomas	[104]
	related	85332	884,7	73	Prkcdbp	involved in cell proliferation mediated by substance P receptor	[112]
	related	56611	868,3	76	Anxa2	correlated with glioma grade and prognosis, its inhibition delays glioma cells migration	[113]
	related	116510	859,6	80	Timp1	expressed in diffuse astrocytomas and involved in neural stem cell maintenance	[114]
	DE	296370	606,2	39	Ctsa	play a role in metastatic dissemination of malignant melanoma	[115]
	DE	289211	592,5	56	Fcgr2b	Fcgamma receptor overexpressed in follicular lymphomas	[116]
	DE	63836	549,6	38	Actn4	role in the survival, motility, and RhoA signaling of astrocytoma cells.	[117]
	DE	25729	457,2	33	Ccne1	Downregulation of Ccne1 by MicroRNA-195 inhibits the proliferation of human glioma cells	[118]
	DE	24908	454,6	26	Dnajb9	suppresses cell death induced by ER stress	[119]
**Ang + PD x Ang 3 h**	related	24525	817,5	70	Kras	Inhibition of Ras is a therapeutic strategy for blocking malignant glioma growth	[120]
	related	25205	799,5	70	Il6st	signal transducer of IL6, interleukin tha promotes glioblastoma cell invasion and angiogenesis	[121]
	related	25687	675,0	65	Cald1	Caldesmon is a cytoskeleton-associated protein involved in glioma neovascularization	[122]
	related	50672	534,3	65	Ednrb	member of a 4-gene signature associated with clinical outcome in high-grade gliomas	[123]
	related	79128	411,5	62	Dab2	downregulation of the tumor supressor gene DAB2 defines a oncogenic pathway in lung cancer	[124]
	DE	116670	280,4	58	Mypt1	participates in Rho-regulated myosin phosphatase required for cell movements and invasion	[125]
	DE	25676	333,1	51	Rasa1	interacts and extensively colocalizes with DLC1 in focal adhesions	[126]
	DE	24577	193,6	49	Myc	Temozolomide suppresses MYC to inhibit progression of human glioblastoma	[127]
	DE	300807	168,4	48	Rora	RORα suppresses breast tumor invasion	[128]
	DE	116663	269,1	45	Dusp6	Dual-specificity phosphatase 6 has tumor-promoting properties in human glioblastomas	[129]
**Ang + PD x Ang 6 h**	related	81778	2241,3	103	S100a10	increased expression in human glioblastoma tumor xenografts expressing EGFRvIII	[130]
	related	65261	1924,3	77	Myo1c	Myo1c facilitates G-actin transport to the leading edge of migrating endothelial cells	[131]
	related	79128	1899,4	95	Dab2	downregulation of the tumor supressor gene DAB2 defines a oncogenic pathway in lung cancer	[124]
	related	85496	1822,7	60	Enpp1	implicated in tumor invasion	[132]
	related	24852	1724,9	79	Tpm4	Tropomyosin that regulates adhesion structures and is a plasma biomarker for lung cancer	[133]
	DE	50719	1330,6	62	Slc7a5	High expression of L-type amino acid transporter 1 in infiltrating glioma cells	[134]
	DE	304577	934,5	60	Ung	uracil-initiated base excision repair pathway occurs in glioblastoma cells	[135]
	DE	54320	928,8	57	Pdpn	Podoplanin is a transmembrane sialoglycoprotein with increased expression in astrocytomas	[136]
	DE	65189	905,9	51	Stk3	participates in the Hippo pathway, that promotes glioblastoma growth	[137]
	DE	362325	896,2	66	Mdfic	transcriptional regulator that modulates Wnt signaling-dependent transcription	[138]

Top-ranked hubs were identified according to their betweenness centrality values and their relevance in tumor biology was determined. The main literature findings on cancer were highlighted for the top five differentially expressed (DE) genes and DE-related genes. Genes that co-express with DE genes (DE-related genes) were included in the networks to verify how DE genes interact with other functionally related genes.

### Evaluation of microarray results by quantitative real-time PCR (qPCR) experiments

**Figure 8:**
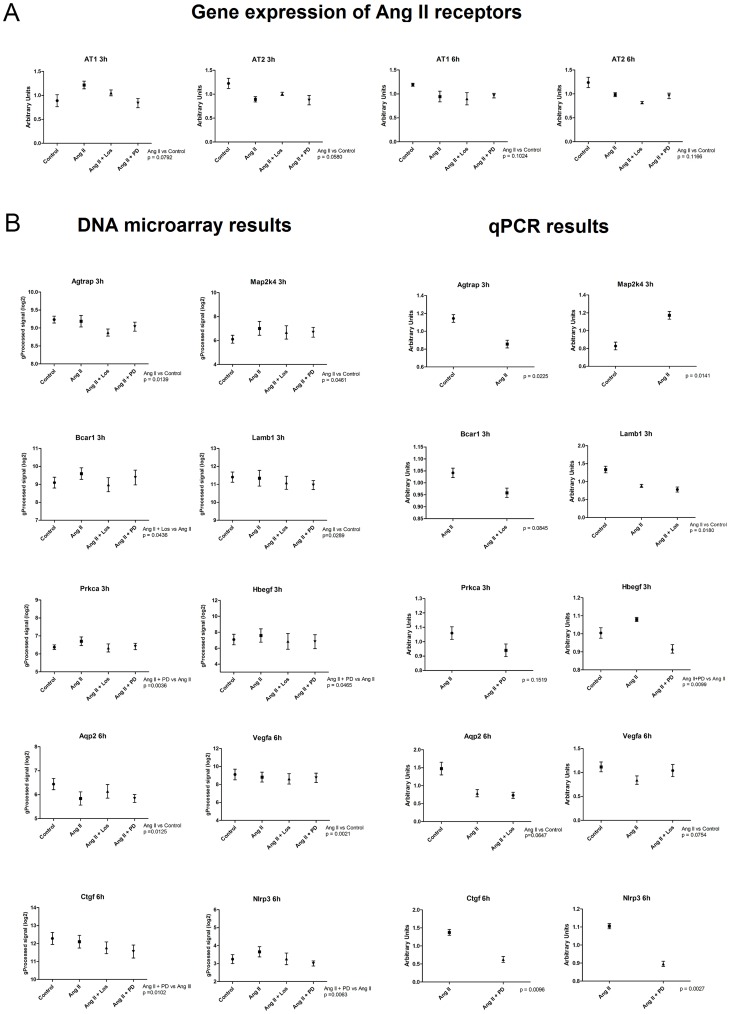
Quantitative PCR (qPCR) experiments for selected genes. A) Gene expression levels of Angiotensin II receptors (Agtr1 and Agtr2) were evaluated by qPCR. B) Technical validation of oligonucleotide microarray data by qPCR of Ang II-regulated genes. The expression of the genes Agtrap, Map2k4, Bcar1, Lamb1, Prkca, Hbegf, Aqp2, Vegfa, Ctgf and Nlrp3 was assessed to confirm gene expression changes identified by oligonucleotide microarray analysis. The gene Gapdh was used as an internal control. The comparison is made between average log2 expression values derived from microarray experiments and arbitrary units obtained from qPCR assays.

The gene expression levels of AT1 and AT2 receptors were evaluated by qPCR analysis. At 3 hours interval, Ang II caused a slight up and down-regulation of AT1 and AT2 receptors, respectively. On the other hand, at 6 hours, Ang II caused a non-significant decrease in the expression of both Ang II receptor subtypes (Figure 8A). The gene expression results of the microarray experiments were confirmed by qRT-PCR of genes regulated by Ang II or its antagonists. Genes were selected according to their fold change values and relevance for cancer biology. Changes in gene expression were confirmed in a range complying with the observed in microarray analysis. Figure 8B shows DNA microarray and qPCR gene expression results.

## Discussion

To gain insights into Ang II effects in gliomas, we analyzed the transcriptomic changes occurring upon Ang II stimulation or AT1 and AT2 inhibition in C6 glioma cells. Interestingly, we found a high number of DE genes with low fold changes at both treatment intervals. This observation is in line with a previous work that investigated Ang II gene targets on glomerulosa cells, revealing widespread effects on gene expression, particularly the rapid induction of numerous transcriptional factors [38]. This transcriptomic pattern is also consonant with the results obtained in a breast cancer cell line stimulated with Ang II [39]. Therefore, it is increasingly evident that Ang II induces a moderate and extensive transcriptional response rather than a strong activation of a limited group of genes.

Venn diagrams showed that Ang II promotes a time-dependent transcriptional response in glioma cells, as different sets of DE genes were observed at each time interval. This effect may be associated with the non-continuous activation of some TFs over time, which is corroborated by the distinct enriched transcription factors observed at each time interval evaluated here. In line with these evidences, a previous study has demonstrated that the kinetics of gene expression changes induced by Ang II can be persistent or transient depending on the cell type [40]. Moreover, acute and chronic Ang II response genes are completely different in adrenocortical cells [41], substantiating this interpretation. Alternatively, these results could be explained by the fact that chronic Ang II treatment regulates the balance between G protein and β-arrestin coupling to Ang II receptors. This regulation consequently changes downstream signaling outcomes and alters the gene expression profiles observed at different intervals [42]. As a matter of fact, alternative G protein-independent pathways such as β-arrestin signaling may be relevant for cancer survival pathways, as β-Arrestin-biased AT1 stimulation activates MAPK pathway [43] and promotes cell survival during acute injury [44].

Our findings likewise indicated that the time-dependent effects of Ang II on glioma cells were altered by the presence of Ang II receptor antagonists. One reason for this is that Ang II exposure modifies the plasma membrane density of its receptors by moving AT1 and AT2 in opposite directions, respectively to the cytosol and to the plasma membrane. However, such receptor translocations are inhibited by Ang II receptor antagonists [45]. Consequently, the ratio of the membrane expression of AT1 and AT2 receptors may be also influenced by the presence of antagonists, taking into consideration the dissociation of antagonist-receptor complexes by Ang II over time. These different ratios would allow Ang II to signal toward distinct downstream pathways, thereby eliciting unique patterns of gene expression depending on the time intervals or antagonists used in the assay.

Notably, gene expression changes induced by Ang II at different time intervals may be relevant for glioma pathogenesis. In accordance with this assumption, our results showed that common genes and transcription factors overrepresented by DE genes at both time intervals were linked to DNA repair, cell cycle control and regulation of tumor suppression and development processes. This anticipates that chronic transcriptome modulation by Ang II may interfere with glioma proliferation in a long-lasting manner. In parallel, another study demonstrated that acute transcriptional changes induced by Ang II influence aortic aneurysm progression [46], suggesting that transient gene expression changes might be also relevant for the protumoral actions of Ang II in glioma cells.

We focused our further transcriptional network analysis on the genes differentially regulated by Ang II and on those that co-express with these DE genes according to GeneMANIA. The systems-level effects of Ang II in glioma cells suggest that this peptide induces transcriptomic changes favoring glioma progression. Such statement is consistent with the presence of network genes whose enriched functions include cell migration, pathways in cancer, mitosis and cell cycle. Additionally, the network-based approach used here enabled the disclosure of protumoral functions of Ang II-regulated genes in glioma cells, through the detection of central network genes (hubs) and their respective overrepresented biological functions. Hub genes were already described to be essential components of biological networks and to play crucial roles in biological systems [47]. In this way, network analysis focusing on the identification of hubs is useful for the prioritization of candidate genes for further analysis [48] and subsequent identification of novel drug targets [49]. Moreover, we functionally enriched hub genes depicted in the networks to unveil which biological functions they participate. This methodology is more advantageous than common gene function enrichment analysis, as it takes into account the interactions among the nodes in the networks, considering a set of genes as an interconnected network. Therefore, the subsequent network-based functional enrichment allows the identification of enriched biological functions among genes that are functionally connected [50]. This is relevant in complex disorders such as cancer that are caused by the interplay between multiple connected genes whose intricate interactions cannot be understood by solely studying individual changes in gene expression [51].

By adopting this approach, we were able to reveal that a significant part of the hub genes participate in signaling pathways frequently deregulated in gliomas [52]–[56]. The diversity of signaling pathways activated by Ang II is in line with previous results showing that Ang II changes the expression of genes involved in many signaling pathways, due to the activation of common second messengers [57]. Most of these genes belong to the cross-talked pathways Ras/Raf/MAPK and PI3K/AKT/mTOR, the two major pathways activated by overexpressed ErbB receptors in glioblastoma cells [58], [59]. These interconnected pathways were previously linked to the trophic effects of Ang II in vascular smooth muscle cells [60]. Moreover, both pathways are stimulated by integrins in the context of focal adhesions, a dynamically regulated process during cell migration [61] and invasion [62]–[64]. Concordantly, two invasion-related functions - focal adhesion and regulation of actin cytoskeleton - were overrepresented by some hub genes identified through network analysis in the different comparisons depicted in Figures 2 to 7.

Noteworthy, AT1 and AT2 inhibitors were both able to down-regulate the expression of hub genes involved in protumoral functions. This result contradicts the standard view in which AT1 and AT2 exert opposite functions in vasoconstriction [65] and cell growth [66]. This paradigm may not apply in a cancer context because tumor cells could also co-opt the signal transduction promoted by AT2 to cooperate with protumoral functions. Such cooperation could be achieved by means of either cumulative genetic mutations or cross-activation of Ang II receptors by other receptors [67]–[69]. Thus, an intriguing hypothesis, still to be fully substantiated, is that both Ang II receptors represent therapeutic targets for intervention in glioma. To date, only the effect of AT1 blockers was evaluated in clinical trials for glioma patients [70]. Therefore, the potential combination of AT1 and AT2 blockers should be tested in animal models of glioma in order to anticipate whether this combination may potentially yield better results in the treatment of those patients.

## Conclusions

In this study, we identified potential molecular mechanisms underlying the correlation between glioma malignancy and positive expression of Ang II receptors. To the best of our knowledge, this is the first work to provide molecular evidence supporting the role of both Ang II receptors in the proliferative and infiltrative behavior of gliomas. Moreover, the hub-based network analysis showed that central genes in the transcriptional networks modulated by Ang II exert functions associated with cell proliferation, migration and invasion, key aspects for glioma progression.

## Methods

### Culture of C6 rat glioma cells and MTT proliferation assay

The C6 rat glioma cell line [71] was used as an in vitro glioma model. The cell line was a gift of Prof. Sueli Kazue Nagahashi Marie from the Neurology Department of the School of Medicine, University of São Paulo. This cell line was obtained from the American Type Culture Collection. They were cultured in Dulbecco's modified Eagle's medium (DMEM) supplemented with 10% fetal bovine serum, 100 Units/ml penicillin and 100 µg/ml streptomycin. Cells were seeded at 10^5^ per well in 96-well plates and allowed to adhere overnight. Four sample replicates were used for each group. They were then incubated at 37°C for 48 h with Ang II. A MTT Proliferation assay (Life Technologies, Carlsbad, US) was carried out to assess C6 cell viability (relative to the untreated control) in response to Ang II treatment. Cells incubated with Ang II had a statistically significant increase in proliferation rate compared to control cells (Student's t test with p<0.0001, data not shown), confirming the capability of Ang II to induce cell proliferation in glioma cells.

### Treatment scheme for oligonucleotide microarray experiments

Cells were seeded in cell culture dishes and incubated at 37°C/5% CO_2_ until becoming confluent. Then, these cells were pre-treated (30 minutes) with either AT1 receptor antagonist (Losartan: 10^−5^ M) or AT2 receptor antagonist (PD123319: 10^−5^ M) followed by Ang II treatment (10^−7^ M) according to the treatment scheme: Group 1 – control; Group 2 – cells only treated with Ang II; Group 3 – cells pre-treated (30 minutes) with Losartan and then treated with Ang II; Group 4 – cells pre-treated (30 minutes) with PD123319 and then treated with Ang II. Ang II, Losartan and PD123319 were obtained from Sigma Chemicals (St Louis, US).

### RNA extraction for oligonucleotide microarray analysis

Total RNA was isolated from samples at 3 and 6 hours intervals using Trizol reagent (Life Technologies, USA) and purified using RNeasy Spin Columns (Qiagen, USA). RNA quantity was determined using a Nanovue spectrophotometer (GE Healthcare, USA). The RNA quality was performed using a 2100 Bioanalyzer with an RNA 6000 Nano kit and Ladder (Agilent Technologies, USA), according to the manufacturer's instructions. The Bioanalyzer produces an electropherogram, which shows the distribution of RNA transcripts in the sample. In an ideal sample, the two peaks of the ribosomal RNA 18 S and 28 S bands are observed, while additional peaks suggest RNA degradation and/or DNA contamination. The 2100 Bioanalyzer Expert software program (version B.02.06.SI418) was used to assign an RNA integrity number (RIN) from 1 to 10, with 1 = degraded, 10 = intact [72]. Only samples with a RNA integrity number (RIN) of 8 or greater were employed.

### RNA amplification and labeling

Agilent's Quick Amp Labeling Kit was used to generate fluorescent cRNA (complementary RNA) for the microarray hybridizations, following the manufacturer's instructions. Briefly, a 700 ηg aliquot of total RNA was reverse transcribed into cDNA. Synthesized cDNA was transcribed into cRNA and labeled with the fluorescent dye Cyanine 3 (Cy3). Labeled cRNA was purified with RNeasy Mini columns (Qiagen). The quality of each cRNA sample was verified by total yield and Cy3 specific activity calculated based on Nanovue spectrophotometer measurements (GE Healthcare).

### Microarray hybridization

Microarray hybridizations were carried out on labeled cRNAs with Cy3 specific activity greater than 9 ρmol Cy3 per µg de RNA. Arrays were incubated at 65°C for 17 h in Agilent's microarray hybridization chambers and subsequently washed according to Agilent's one-color microarray-based gene expression analysis protocol (Version 5.7, March 2008). Gene expression profiles were evaluated using Agilent whole rat genome 4×44K oligonucleotide microarrays.

### Data acquisition

Hybridized slides were scanned at 5 µm resolution using an Agilent G2505B DNA microarray scanner. Default settings were modified to scan the same slide twice at two different sensitivity levels (XDR Hi 100% and XDR Lo 10%). The two linked images generated were analyzed together and data were extracted and background subtracted using the standard procedures contained in the Agilent Feature Extraction (FE) Software version 9.5.1. The software returns a series of spot quality measures to assess the reproducibility and the reliability of spot intensity estimates. These parameters are summarized in a quality control report and were evaluated in order to support the high quality of the data acquired.

### Data processing and analysis

The R statistical environment (http://www.r-project.org) was used to filter and analyze the data. The mean of the probes for each gene was calculated, genes with missing values were removed and then the signal intensities were log2 transformed. Subsequently, data was normalized using the Löwess normalization method [73] to correct intensity-dependent ratio bias between the arrays. These logarithmic normalized values were used to perform the statistical analyses. To identify which genes were significantly differentially expressed, t-tests (p<0.05) were performed in the following comparisons: Ang II x Control (3 h); Ang II x Control (6 h); Ang II+Los x Ang II (3 h); Ang II+Los x Ang II (6 h); Ang II+PD123319 x Ang II (3 h); Ang II+PD123319 x Ang II (6 h). Microarray data set supporting the results of this article is available in GEO public database (http://www.ncbi.nlm.nih.gov/geo), under accession number GSE47529.

### Functional enrichment analyses

The differentially expressed genes found at 3 and 6 hours intervals were used to carry out extensive analysis of functional categories, i.e., gene ontology (GO) terms [74] and Kyoto Encyclopedia of Genes and Genomes (KEGG) pathways [75]. We aimed at clarifying whether certain gene clusters are enriched with particular over-represented functional categories. This categorization was made using FunNet (http://www.funnet.ws) as an intermediary tool. FunNet performs a functional profiling of gene expression data, identifying overrepresented biological themes in the microarray dataset using GO and KEGG databases [76]. The common differentially expressed genes across the comparisons were identified using the Gene List Venn Diagrams software [77]. Finally, transcription factors (TFs) upstream to differentially expressed genes were identified using Enrichr, a bioinformatics tool that retrieves molecular information from transcription factor databases and defines transcription factors statistically enriched from gene lists [78]. We used the p-value calculated by Enrichr for ranking enrichment results and were considered significant the top 100 transcription factors with p<0.05. In addition, only the TFs overrepresented in both ChEA and Transfac databases were used for further analysis.

### Network analysis of transcriptional changes

Transcriptional network analysis was performed to evaluate the network representation of the molecular relationships between DE genes. GeneMANIA [79], a Cytoscape plugin, was used to predict DE gene interactions and to expand the networks with functionally similar genes, using available genomics and proteomics data. Networks were generated using only information derived from the co-expression category [80]. Node centrality values were calculated with CentiScaPe [81] in order to determine their hierarchy in the respective biological networks. We used the centrality betweenness and degree indexes, which correspond, respectively, to the number of links incident upon a node and to the number of shortest paths that passes through a specific node to connect directly or indirectly two other nodes. GraphPad Prism software (GraphPad, San Diego, CA, USA) was used to build scatter plots showing the correlation between each node degree and betweenness. Scatter plots allowed the selection of the 25 DE genes and 15 related genes with highest centrality values in each network. Finally, networks showing the interactions between these top-ranked genes were obtained using Cytoscape. Node size was proportional to betweenness centrality values. In order to retrieve literature information pertaining to each gene we used GenClip, a bioinformatics tool that searches for genes related to keywords based on up-to-date literature profiling [82].

### Validation of microarray by real-time qRT-PCR

To validate the results of microarray analysis, the differential expression of representative genes in the microarray analysis was confirmed using the same RNA samples that were used for microarray, by qRT-PCR amplification. For cDNA synthesis, an aliquot of 0,5 µg of total RNA from each sample was incubated with 1 µL of Oligo(dT) 0,05 µg/µL, 1 µL de SuperScript II Reverse Transcriptase and completed with 20 µL of DEPC treated water. For polymerase chain reaction (PCR) to amplify cDNA, QuantiFast SYBR Green PCR Kit (Qiagen) was used according to the manufacturer's instructions in a final volume of 25 µL per reaction. The following primers (10 pmol/µL) were designed using Primer 3 software (23) and used for qRT-PCR: Agtr1 sense (CCATCGTCCACCCAATGAAG) and antisense (GTGACTTTGGCCACCAGCAT), Agtr2 sense (AATTACCCGTGACCAAGTCTTG) and antisense (ATACCCATCCAGGTCAGAGCAT), Prkca sense (ATGCTCATGTTTCCAGTCTGC) and antisense (CTGATAGAGTGCCAGTGTGTGG), Agtrap sense (CAAAGAAGACAAGAAGCCCAAG) and antisense (AGCGCTCTACCACTGAGCTAAA), Map2k4 sense (GTGGACAGCTCGTGGACTCTAT) and antisense (TCATACCCTTGTCTTGATGCAC), Hbegf sense (GGAGAGTGCAGATACCTGAAGGA) and antisense (GTCAGCCCATGACACCTCTGT), Ctgf sense (AAGAAGACTCAGCCAGACC) and antisense (AGAGGAGGAGCACCAAGG), Nlrp3 sense (CAGACCTCCAAGACCACGACTG) and antisense (CATCCGCAGCCAATGAACAGAG), Bcar1 sense (GCACACAGCAAGTTTGTCATTC) and antisense (GCTGTAGTGGGTCACTTTGCTT), Aqp2 sense (CTGGTGCTGTGCATCTTTGC) and antisense (ATGGAGCAACCGGTGAAAT), Lamb1 sense (GCGTAAAGCTGCCCAGAACTCTG) and antisense (TCCTCCTGGCATCTGCTGACTC), Vegfa sense (GCCCATGAAGTGGTGAAGTT) and antisense (TATGTGCTGGCTTTGGTGAG), and Gapdh sense (GACATGCCGCCTGGAGAAAC) and antisense (AGCCCAGGATGCCCTTTAGT), this last used as a housekeeping gene. The amplification protocol design was: initial denaturation step at 95°C for 5 min followed by 40 cycles using Applied Biosystems 7300 Real-Time PCR System. Each cycle included a denaturation step at 95°C for 15 seconds and a primer-annealing/elongation step at 60°C for 30 seconds. The relative standard curve method was used for quantification of gene transcription between the groups evaluated. For all PCR reactions, dissociation curves were constructed in order to verify the amplification reaction specifity and confirm the absence of primer dimer formation. Statistical analysis was carried out using one-way paired t-tests between selected experimental groups using a significant threshold of p<0.10.

## Supporting Information

File S1 **Tables S1 to S12: Differentially expressed (DE) genes for each comparison and their respective enriched biological functions.** DE genes were functionally enriched using Gene Ontology (GO – Biological Processes) and Kyoto Encyclopedia of Genes and Genomes (KEGG) databases, through FunNet bioinformatics tool.
(PDF)

File S2 **Table S13: DE gene's p-value and fold changes for all comparisons at 3 hours interval after Ang II treatment.**
(XLSX)

File S3 **Table S14: DE gene's p-value and fold changes for all comparisons at 6 hours interval after Ang II treatment.**
(XLSX)

File S4 **Table S15: Venn diagrams showing the overlapping differentially expressed (DE) genes across the experimental comparisons.** Venn diagrams were constructed using all experimental comparisons in order to identify DE genes regulated by Ang II at both 3 or 6 hours intervals, and genes whose expression is altered by Ang II and by the presence of Ang II and its antagonists. Genes encompassed in these overlaps are called common genes.
(XLSX)

File S5 **Table S16: Venn diagrams showing the enriched transcription factors (TFs) overlapped among the experimental comparisons.** Venn diagrams were constructed using the top 100 enriched TFs disclosed at both ChEA and Transfac databases in order to identify significant enriched transcription factors across the experimental comparisons.
(XLSX)
