# Supplementary material for: Transcriptional Network Analysis Reveals that AT1 and AT2 Angiotensin II Receptors Are Both Involved in the Regulation of Genes Essential for Glioma Progression
Source: PLoS One. 2014 Nov 3;9(11):e110934. doi: 10.1371/journal.pone.0110934 (PMC4217762; doi:10.1371/journal.pone.0110934)
Supplement: File S1 — Tables S1 to S12: Differentially expressed (DE) genes for each comparison and their respective enriched biological functions. DE genes were functionally enriched using Gene Ontology (GO – Biological Processes) and Kyoto Encyclopedia of Genes and Genomes (KEGG) databases, through FunNet bioinformatics tool. (PDF) [file pone.0110934.s001.pdf]

Table S1. KEGG categories and respective enriched genes regulated by Ang II at 3 hours interval

KEGG category	Up-regulated Genes
**MAPK signaling pathway**	Mapk1, Cdc25b, Akt1, Kras, Map2k4, Mapkapk2, Mecom, Map2k5, Ppp3r1, Elk1, Rras, Ppp5c, Gna12
**Chemokine signaling pathway**	Shc3, Mapk1, Akt1, Kras, Cxcl10, Cxcl12, Gnai3, Prex1, Gnb2
**Regulation of actin cytoskeleton**	Mapk1, Apc, Kras, Myl9, Rras, Fgd1, Pdgfc, Gna12, Git1
**Tight junction**	Akt1, Kras, Magi3, Gnai3, Myl9, Inadl, Rras, Pard6b, Llgl1
**ErbB signaling pathway**	Shc3, Mapk1, Akt1, Kras, Map2k4, Elk1, Nck2
**Neurotrophin signaling pathway**	Shc3, Mapk1, Akt1, Kras, Mapkapk2, Map2k5, Sh2b1
**Ubiquitin mediated proteolysis**	Keap1, Ube2e3, Pias1, Mgrn1, Klhl13, Anapc10, LOC68922
**Insulin signaling pathway**	Shc3, Mapk1, Akt1, Kras, Elk1, Rhoq
**Chronic myeloid leukemia**	Shc3, Mapk1, Akt1, Kras, Mecom, E2f1
**Cell cycle**	Cdc25b, Mcm5, Mcm6, Tfdp2, Anapc10, E2f1
**Glioma**	Shc3, Mapk1, Akt1, Kras, E2f1
**VEGF signaling pathway**	Mapk1, Akt1, Kras, Mapkapk2, Ppp3r1
**Gap junction**	Mapk1, Kras, Gnai3, Map2k5, Pdgfc
**KEGG category**	**Down-regulated Genes**
**Protein processing in endoplasmic reticulum**	Stub1, Ganab, Bag1, Tram1, Ckap4, Man1b1, Wfs1
**Axon guidance**	Sema3c, Nfat5, Efn4, Ephb6, Unc5a, Slit1
**ECM-receptor interaction**	Lama5, Cd47, Col1a1, Col5a3
**TGF-beta signaling pathway**	Ep300, Gdf7, Ltbp1, Tgfb2
**PPAR signaling pathway**	Dbi, Slc27a4, Scd4, Slc27a1
**mTOR signaling pathway**	Rps6ka2, Ins1, Tsc2
**Notch signaling pathway**	Ep300, Ncstn, Psen2
**Aldosterone-regulated sodium reabsorption**	Ins1, Prkca, Scnn1a
**N-Glycan biosynthesis**	Ganab, Alg11, Man1b1

Table S2. Biological processes (GO) and respective enriched genes regulated by Ang II at 3 hours interval

Biological Process	Up-regulated Genes
cell cycle	Apc, Ccng1, Nucks1, Rab11b, Sept9, Rgs2, Mapk1, Cdc25b, Cgref1, Mecom, Thap1, Specc1l, Ankrd54, Lzts, E2f1, Pdcd6ip
cell division	Cdc25b, Ccng1, Gnai3, Specc1l, LZts2, Pdcd6ip, Sept9
cell growth	Akt1, Cxcl12, Map2k5, Taf9
protein transport	Nutf2, Sec13, Atp6v1e1, Ipo9, Chchd4, Pex5, Chmp1a, Pdcd6ip, Exoc5, Ykt6, Atp6v1a, Rab11b
intracellular signal transduction	Shc3, Mapk1, Akt1, Def8, Dnmbp, PRerx1, Blnk, Grb15,Sh2b1
small GTPase mediated signal transduction	Mapk1, Kras, Rnd2, Rras, Arl6, Rab11b, Rhoq, Gnai3, Gfm2, Gna12
protein folding	Fkbp4, Ranbp2, Ppil4, Dnajc11, Ptges3, Grpel1, St13
protein ubiquitination / polyubiquitination	Akt1, Mgrn1, Klhl13, Rnf187, Zfp364, March2, Ube2e3, Ube2v1, Mgrn1, Rnf187, LOC689226
actin cytoskeleton organization	Kras, Fhl3, Fgd1, Llgl1, Cap1, Rhoq
MyD88-dependent toll-like receptor signaling pathway	Mapk1, Map2k4, Mapkapk2, Ube2v1, Elk1
Wnt receptor signaling pathway	Wif1, Apc, Ldb1, Lzts2
response to DNA damage stimulus	Mapk1, Apc, Apbb1, Paxip1, Obfc2b
response to ionizing radiation	Cxcl10, Vcam1, Paxip1, Obfc2b
**Biological Process**	**Down- regulated Genes**
ion transport	Steap3, Tmem37, Scnn1a, Kcnh3, Kcnk3, Gabrd, Slc22a18, Cacna1i, Slc22a23, Slc12a6
brain development	Ptprg, Dbi, Cdk5rap1, Nkx2-1, Cdk5rap2, Kcnk3, Bcr, Slc7a11, Slit1, Hrh3
protein transport	Steap3, Aagab, Dnm2, Mcoln1, Cox18, Apoc2, Col1a1, Lca5, Tram1, Psen
heart development	Rps6ka2, Sox9, Ep300, Gja1, Tsc2, Oxt, Sema3c, Sox18, Tgfb2
negative regulation of cell proliferation	Sox9, Gja1, Grm4, Igfbp3, Prkca, Tsc2, Serpine2, S2f7, Tgfbp2
negative regulation of apoptosis	Sox9, Grm4, Bag1, Cln8, Tex11, Psen2, Tgfb2, Wfs1
response to hypoxia	Ep300, Pnmt, Tsc2, Kcnk3, Agtrap, Adsl, Psen2, Tgfbp2
axon guidance	Gdf7, Nkx2-1, Neurog2, Unc5a, Slit1, Tgfb2
protein folding	Ganab, Bag1, RGD1565752, Dnajc17, Fxbp10, Man1b1
negative regulation of cell growth	Dnajc2, Serpine2, Ndufa13, Slit1, Tgfb2
cell migration	Lama5, Cd47, Lamb1, Mdk, Tgfb2

Table S3. KEGG categories and respective enriched genes regulated by Ang II at 6 hours interval

KEGG category	Up-regulated Genes
**Cell cycle**	Chek2, Ccna2, Bub1b, Ccnb1, Ywhaq, Skp2, Smc3, Mcm4, Hdac1, Anapc1, Stag2, Cdc7, MGC112830, Anapc10, Cdk2, Cdc26, Cdkn2c, Cdkn2d, Cdc20
**Pathways in cancer**	Mapk1, Smo, Ctbp1, Skp2, Hdac1, Pias1, Stk36, Col4a2, Cdk2, Tcf7, Mapk9, Ccdc6, Grb2, Mlh2, Sos2
**RNA transport**	Nup107, Eif4e, Eif4a1, Thoc3, Ncbp1, Pabpc4, Sumo1, RGD1306195, Nup205, Eif4e2, Gemin7, Sumo2, Thoc4
**MAPK signaling pathway**	Mapk1, Mapk4k3, Hspa8, Hspa1l, Ntf4, Map2k4, Pak2, Elk1, Mapk9, Chp, Grb2, Sos2
**Focal adhesion**	Mapk1, Capn2, Pak2, Vcl, Col4a2, Elk1, Vasp, Itga8, Mapk9, Grb2, Sos2
**Insulin signaling pathway**	Mapk1, Eif4e, Elk1, Eif4e2, Prkag2, Mapk9, Calm2, Grb2, Sos2
**Protein processing in endoplasmic reticulum**	Nploc4, Hspa8, Hspa1l, Hsph1, Capn2, Man1a2, Ube2j2, Mapk9, Eif2ak2
**Spliceosome**	Hspa8, Hspa1l, Thoc3, Snrpd1, Ncbp1, lsm6, Pcbp1, Plrg1, Thoc4
**Ubiquitin mediated proteolysis**	Skp2, Ube2q1, Ube2j2, Pias1, Anapc1, Anapc10, Ube2l3, Cdc26, Cdc20
**Proteasome**	Psme3, Pomp, Psma1, Psmd14
**ErbB signaling pathway**	Mapk1, Map2k4, Pak2, Abl2, Elk1, Mapk9, Grb2, Sos2
**Neurotrophin signaling pathway**	Mapk1, Ywhaq, Ntf4, Nfkbie, Mapk9, Calm2, Grb2, Sos2
**p53 signaling pathway**	Chek2, Ccnb1, Ei24, Cdk2, Zmat3
**Adherens junction**	Mapk1, Yes1, Vcl, Ctnnd1, Tcf7
**Chronic myeloid leukemia**	Mapk1, Ctbp1, Hdac1, Grb2, Sos2

KEGG category	Down-regulated Genes
**Parkinson's disease**	Cox6a2, Cyc2, Ndufs2, Ube2g2, Ube2l6, Ndufa12, Ndufa6, Ndufv3, Ndufa3
**Mineral absorption**	Slc31a1, Slc39a3, Slc26a6, Hmox2
**Cytosolic DNA-sensing pathway**	Ripk3, Ifna4, RGD1562552, Polr3g
**Pantothenate and CoA biosynthesis**	Coasy, Ppcs, Enpp3
**Protein export**	Srp19, Srpr, Sec61g
**Riboflavin metabolism**	Acp5, Enpp3

Table S4. Biological processes (GO) and respective enriched genes regulated by Ang II at 6 hours interval

Biological Process	Up-regulated Genes
**protein phosphorylation**	Chek2, Nek2, Wnk1, Mapk1, Cdk5, Map4k3, Camk1, Cdk12, YEs1, Ccnb1, Dyrk1a, Map2k4, Pak2, Sgk1, Tbk1, Lrrk2, Stk36, Abl2, Pxk, Mst4, Nek6, Spag9, Cdc7, Vrk1, Cdk2, Pik3r4, Mapk9, Eif2ak2
**negative regulation of apoptosis**	Dffa, Il6r, Serbp1, Glp1r, Smo, Fnta, Accn1, Fignl1, Gata6, Hells, Htt, Skp2, Sgk1, Hdac1, Apbb2, Serpinb9, Pea15a, Taf9, Rtn4, Twist1
**positive regulation of apoptosis**	Dffa, Cdk5, Aldh1a3, Map2k4, Gata6, Skp2, Ing4, Sav1, Apbb2, RGD1309922, Top2a, Mapk9, Eif2ak2
**proteasomal ubiquitin-dependent protein catabolic process**	Bub1b, Ccnb1, Psme3, Ube2v2, Psmd5, Psma1, lrrk2, Sumo1, Fbxl5, Psmd14, Anapc1, Anapc10, Trim13, Cdc26, Taf9, Cdc20, Ppp2r5c, Gipc1
**negative regulation of cell proliferation**	Phb2, Cd9, Igfbp5, Tinf2, Ing4, Hdc1, Sav1, Lrrk2, Brip1, Cdkn2c, Eif2ak2, Dlc1, Slc9a3r1, Mfn2, Inppl1, Ppp2r5c
**cell cycle**	Ccna2, Clasp2, Mapk1, Cdk5, Kif2c, Smc3, Arhgef2, Nek6, Spast, Cdk2, Cdc26, Calm2, Cdc20, Mlh1
**cell division**	Ccna2, Clasp2, Cdk5, Kif2c, Ccnb1, Smc3, Arhgef2, nek6, Top2a, Spast, Cdk2, Cdc26, Cdc20, Top1
**nervous system development**	Myo1b, Camk1, Dyrk1a, Accn1, Ube2v2, Dpf3, Lrrk2, Ophn1, Spast, Kctd11, LOC606294, Rtn4, Nrn1, Ppp1r9b
**DNA repair**	Polk, Wrnip1, Ube2v2, Polq, Fbxo18, Rbbp8, Fto, Smc3, Pole, Rev3l, Recql, Rnf168, Mlh1, Parg
**actin cytoskeleton organization**	Cdk5, Mk1, Sipa1l1, Coro1b, Capzb, Abl2, Ophn1, Ehd2, Vasp, Dlc1, Slc9a3r1, Cap1, Capza1
**DNA replication**	Wrnip1, Map2k4, Polq, Mcm4, Ing4, Pole, Rev3l, Recql, Cdc7, Cdk2, Helb, Top1
**protein ubiquitination**	Rnf34, Skp2, Rnf2, Fbxl5, Anapc1, Anapc10, Rfws3, Ube2l3, Trim13, Cdc26, Rnf168, Ccnb1, Psme3, Psmd5, Psma1, lrrk2, Psmd14, Tbc1d7, Cdc20
**mitosis**	Clasp2, Kif2c, Ccnb1, Jtb, Smc3, Arhgef2, Nek6, Cdk2, Cdc26, Cdc20
**RNA splicing**	Cdk13, Snrpd1, Cd2bp2, Ncbp1, Hnrnpa2b1, Srek1, Plrg1, Thoc4, Polr2f

Biological Process	Down- regulated Genes
apoptosis	Psmd9, Acsbg1, Ripk3, Prkcb, Cycs, Aqp2, Ncstn, Psmg2, Actc1, Fastk, Arhgef4, Ciapin1, Plekhf1, Trim39, Traf2, Aen, Itgb3bp, Scrip, Pdcd2, Dapk3, Aifm1, Plg
protein transport	Pgap2, Arfgap1, Abra, Vps36, Snx17, Rabif, Chmp2b, Chmp7, Efcab4b, Arfgap3, Gorasp1, Vps33b, Pex14, Snap23, Scamp2, Vps25, LOC681989, Fxc1, Rab27b, Sec31a
intracellular protein transport	Vamp1, Abra, Ipo4, Napg, Ipo7, Srpr, Snupn, Tom1l2, Tom1, Sar1a, Xpo5, Scamp2, LOC683402
protein ubiquitination	Zfp91, Ube2g2, Rnf146, Uhrf2, Traf2, Tri32, Klhl21, Cbll1, Mdm2, Ube3a, Herc6, Anapc13
ubiquitin-dependent protein catabolic process	Usp2, Usp4, Usp15, Rnf146, Uhrf2, Trim32, Mdm2, Ube3a, Herc6, Usp48
response to oxidative stress	Abcc1, Coq7, Cycs, Ndufs2, Ndufa12, Ppp1r15b, Lias, Ndufa6, Hmox2
vesicle-mediated transport	Arfgap1, Vamp1, Sar1a, Vamp4, Arfgap3, Vps33b, Snap23, LOC683402, Sec31a
protein targeting	Optn, Srp19, Strada, Mdm2, Srpr, Xpo5, Pmpcb, Pex14, Fxc1
positive regulation of cell migration	Abcc1, Acp5, Trim32, Cbll1, Srpx2, Cxcl16, Ager, Vegfa
cytoskeleton organization	Cdc42bpa, Uxt, Bbs4, Tekt4, Tubgcp2, MAST1, Ablim2, Trpv4, Rhoq

Table S5. KEGG categories and respective enriched genes regulated by Ang II via AT1 receptor at 3 hours interval

KEGG category	Up-regulated Genes
**Metabolic pathways**	Ndst2, Hsd17b1, B3gnt1, Polrf3f, Etnk1, Rdh12, Chpf, Pigu, Alg11, Alg9, Rfk, Rpe
**Lysosome**	Mcoln1, Aga, Abca2
**Acute myeloid leukemia**	Myc, Rps6kb2
**Steroid hormone biosynthesis**	Sts, Hsd17b1
**N-Glycan biosynthesis**	Alg11, Alg9
**KEGG category**	**Down-regulated Genes**
**Cell cycle**	Chek2, Cdc25b, Rp53, Anapc5, Mcm5, Smc1a
**p53 signaling pathway**	Chek2, Tp53, Tsc2
**Amino sugar and nucleotide sugar metabolism**	Gale, Amdhd2, Gmppa
**beta-Alanine metabolism**	Acadm, Aldh2
**Propanoate metabolism**	Acadm, Aldh2
**Glycine, serine and threonine metabolism**	Shmt2, Gcat
**Vasopressin-regulated water reabsorption**	Dctn6, Dynll1
**DNA replication**	Mcm5, Pola1

Table S6. Biological processes (GO) and respective enriched genes regulated by Ang II via AT1 receptor at 3 hours interval

Biological Process (GO)	Up-regulated Genes
protein transport	Lman1, Unc50, Mcoln1, Tram1, Atg9a
positive regulation of apoptosis	Jak2, Myc, Tlr4, Stk4
steroid metabolic process	Lcat, Sts, Hsd17b1, Scap
inflammatory response	Tnfrsf1b, Tnfrsf4, Tlr4, Jak, Ccl24, Zyx, Cd276
tumor necrosis factor-mediated signaling pathway	Tnfrsf1b, Jak2, Tnfrsf4
positive regulation of cell migration	Jak2, Ccl24, Lamb1
intracellular protein kinase cascade	Jak2, Stk4

Biological Process (GO)	Down-regulated Genes
cell cycle / division and mitosis	Cdc25b, Avpi1, Ppp1c, Anapc5, Kifc1, Mtus1, RGD1306730, Smc1a, Arl2, Mlh1
regulation of cell cycle	Cdc37, Tp53, Tsc2, Bop1
DNA repair	Fbxo18, Kctd13, Smc1a, Mlh1, Pola1
carbohydrate metabolic process	Gale, Ppp1c, Amdhd2, Chst5
protein homooligomerization	Acadm, Tsc2, Kctd13, Shmt2
cell migration	Bcar1, Kctd13, Gsk3a, Bambi
negative regulation of signal transduction	Tsc2, Erbb3, Gsk3a, Rfng
DNA damage response, signal transduction resulting in induction of apoptosis	Chek2, Tp53, Mlh1
circadian rhythm	Nfil3, Erbb3, Arntl
insulin receptor signaling pathway	Eif4ebp1, Bcar1, Gsk3a
anaphase-promoting complex-dependent proteasomal ubiquitin-dependent protein catabolic process	Psmd9, Psme3, Anapc5
neuroblast proliferation	Tp53, Racgap1, Sox2
TOR signaling cascade	Eif4ebp1, Tsc2
positive regulation of epithelial to mesenchymal transition	Wwtr1, Bambi
cytokinesis	Incenp, Racgap1
Rho protein signal transduction	Kctd13, Cdc42ep1
negative regulation of Wnt receptor signaling pathway	Tsc2, Sox2

Table S7. KEGG categories and respective enriched genes regulated by Ang II via AT1 receptor at 6 hours interval

KEGG category	Up-regulated Genes
**RNA transport**	Eif4e, Ube2i, Pop4, Nup35, Gemin6, Pigz, Gemin2
**Cell cycle**	Chek1, Ccne1, Cdc45, Mad2l1, Gadd45b
**Peroxisome**	Pxmp4, Pex19, Eci2, Pex11b
**Pyrimidine metabolism**	Uck1, Cmpk2, Polr3k, Polr3gl
**p53 signaling pathway**	Chek1, Ccne1, Gadd45b
**Proteasome**	Psmb9, Psbm4, Psmb1
**Riboflavin metabolism**	Acp5, Acp6
**Homologous recombination**	Rad54b, Rpa2
**RNA polymerase**	Polr3k, Polr3gl
**KEGG category**	**Down-regulated Genes**
**Focal adhesion**	Bcar1, Ptk2, Itgb4, Pak1, Myl9, Flnb, Itgb6, Actn4, Vegfa
**Regulation of actin cytoskeleton**	Bcar1, Ptk2, Itgb4, Pak1, Myl9, Itgb6, Iqgap1, Actn4
**Chemokine signaling pathway**	Plcb1, Stat3, Bcar1, Ptk2, Pak1, Gngt2
**Lysosome**	Ctsh, Cdig2, Gga2, Ctsa, Abcb9
**Axon guidance**	Ptk2, Pak1, Sema3c, Sema3f, Slit2
**PPAR signaling pathway**	Acsl1, Scd4, LOC681458, Gk
**Adherens junction**	Smad2, Ssx2ip, Iqgap1, Actn4
**Aminoacyl-tRNA biosynthesis**	Aars, Farsb, Wars
**Biosynthesis of unsaturated fatty acids**	Scd4, LOC681458
**Vitamin digestion and absorption**	Tcn2, Cubn

Table S8. Biological processes (GO) and respective enriched genes regulated by Ang II via AT1 receptor at 6 hours interval

Biological Process (GO)	Up-regulated Genes
protein ubiquitination	Psmb9, Arrb2, Ube2i, Ube2t, Psmb4, Rnf168, Psmb1, Arrb2, Mad2l1, Birc5
proteasomal ubiquitin-dependent protein catabolic process	Psmb9, Arrb2, Ube2i, Mad2l1, Jkamp, Psmb4, Psmb1
mitotic cell cycle	Eif4e, Psmb9, Mad2l1, Psmb4, Birc5, Psmb1
DNA repair	Chek1, Gen1, Alkbh2, Rpa2, Rnf168
regulation of cell growth	Cd44, Exosc9, Ccdc85b, Ulk1, Zfp639
cell division	Ube2i, Ccne1, Cdc45, Spast, Birc5
cellular process involved in reproduction	Sep15, Arrb2, Nkx2-1, Syt6, Zfp639
cell cycle process	Psmb9, Mad2l1, Psmb4, Birc5, Psmb1
response to peptide hormone stimulus	Prss8, Aqp2, Prlh, Birc5
**Biological Process (GO)**	**Down-regulated Genes**
signal transduction	Chd4, Cdh13, Grin2c, Ngfr, Il1rl1, Itpr3, Xpr1, Aars, Srebf2, Arhgap28, Fert2, Scara5, Ngf, Iqgap1, Vapb, Arhgap10
cell differentiation	Egr2, Klf4, Sox9, Ngfr, Plcb1, Ptk2, Jag1, Clip3, Cd276, Slit2, Zmiz1, Sohlh1, Srf, Ascl1, Dhh
response to organic substance	Abcc4, Cdh13, Stat3, Acsl1, Fosl1, Ptk2, Sdc2, Smad2, Pak1, Srebf2, Ngf, Slit2, Pea15a, Hspd1, GK
regulation of cell proliferation	Nrg1, Sox9, Abcc4, Cdh13, Ngfr, Irs1, Ptk2, Ngf, Zmiz1, Sox4, Apln, Nolc1, Vegfa, Nr2f2, Klf4, Jag1
negative regulation of apoptosis	Nrg1, Sox9, Cdh13, Ngfr, Dnajb9, Ptk2, Lrp1, Ngf, Pea15a, Sox4, Hspd1, Ascl1, Vegfa
response to stress	Adh5, Ubqln1, Dnajb9, Sdc2, Aars, Pak1, Scara5, Sox4, Vapb, Actn4, Hspd1, Gk, Vegfa
regulation of signal transduction	Klf4, Avpi1, Cdh13, Ngfr, Irs1, Jag1, Smad2, Pak1, Ngf, Sulf2, Slit2, Ascl1, Vegfa
cell surface receptor linked signaling pathway	Plcb1, Spn, Stat3, Acsl1, Bcar1, Gfra1, Il1rap, Il1rl1, Itgb4, Fcgr2b, Itgb6, Slit2
cell adhesion	Nrg1, Cdh13, Bcar1, Ptk2, Itgb4, Fert2, Pcdh9, Ssx2ip, Itgb6, Omd

Table S9. KEGG categories and respective enriched genes regulated by Ang II via AT2 receptor at 3 hours interval

KEGG category	Up-regulated Genes
**Purine metabolism**	Gucy1b2, Pfas, Polr2i
**Steroid hormone biosynthesis**	Ugt1a6, Cyp1b1
**Retinol metabolism**	Ugt1a6, Dhrs3
**Metabolism of xenobiotics by cytochrome P450**	Ugt1a6, Cyp1b1
**Drug metabolism - other enzymes**	Ugt1a6, Ces2a
**N-Glycan biosynthesis**	Ganab, Man1c1

KEGG category	Down-regulated Genes
**Pathways in cancer**	Pik3ca, Itgb1, Myc, Prkca, Fgf9, Axin2, Hdac1, Cbl, Birc5, Sos2
**MAPK signaling pathway**	Dusp6, Myc, Prkca, Fgf9, Rasa1, Pak2, Ppp3r1, Rras, Sos2
**Regulation of actin cytoskeleton**	Ppp1r12a, Pik3ca, Itgb1, Fgf9, Pak2, Itga4, Rras, Sos2
**ErbB signaling pathway**	Pik3ca, Myc, Prkca, Hbegf, Areg, Pak2, Cbl, Sos2
**Focal adhesion**	Ppp1r12a, Pik3ca, Itgb1, Prkca, Pak2, Itga4, Sos2
**Axon guidance**	Itgb1, Rasa1, Pak2, Ppp3r1
**VEGF signaling pathway**	Pik3ca, Prkca, Ppp3r1

Table S10. Biological processes (GO) and respective enriched genes regulated by Ang II via AT2 receptor at 3 hours interval

Biological process (GO)	Up-regulated Genes
cell differentiation	Igfbp3, Gpsm1, Ggn, Sox18, Lrp6, Nr0b1, Mdk
angiogenesis	Ecm1, Cyp1b1, Ccl24, Sox18, Pnpla6
response to peptide hormone stimulus	Ugt1a6, Pnmt, Lrp6, Nr0b1
embryo development	Foxn3, Ccno, Foxj2, Fzd2
regulation of sequence-specific DNA binding transcription factor activity	Lrp6, Foxn3, Foxj2, Nr0b1
vasculature development	Cypb1, Sox18, Fzd2
canonical Wnt receptor signaling pathway	Lrp6, Fzd2, Bambi

Biological process (GO)	Down-regulated Genes
cell differentiation	Dusp6, Bzw2, Fgf9, Snai2, Rasa1, Axin2, Areg, Ppp3r1, Rora, Runx2, Sh2b3, Fhl2, Ctgf, Dhh
negative regulation of apoptosis	Pik3ca, Myc, Serbp1, Rasa1, Zc3hc1, Hdac1, Api5, Pea15a, Cbl, Birc5, Pim3
positive regulation of cell proliferation	Itgb1, Myc, Prkca, Hbegf, Fgf9, Areg, Hdac1, Etv5, Aggf1, Runx2, Ctgf
ossification	Mmp13, Fgf9, Snai2, Axin2, Areg, Impad1, Nab2, Runx2, Fhl2, Ctgf
negative regulation of cell proliferation	Gtpbp4, Itgb1, Prkca, Emd, Eif2ak1, Axin2, Hdac1, Eif2ak2
cell adhesion	Cdh17, Itgb1, Rasa1, Tnfrsf12a, Aggf1, Itga4, Ctgf, Cdhr1
canonical Wnt receptor signaling pathway	Emd, Fgf9, Snai2, Axin2, Hdac1, Lats2, Cyld
regulation of signal transduction	Dusp6, Zfp91, Prkca, Fgf9, Slc7a1, Hdac1, Runx2
regulation of cell proliferation	Myc, Prkca, Hbegf, Emd, Fgf9, Etv5, Eif2ak2
aging	Lonp1, Prkca, Eno3, Canx, Ctgf, Tfrc
dephosphorylation	Dusp6, Ppp1r12a, Acp2, Ppp3r1, Impad1, Pdp1
regulation of mitotic cell cycle	Myc, Cyld, Brd4, Birc5, Pim3, LOC690728

Table S11. KEGG categories and respective enriched genes regulated by Ang II via AT2 receptor at 6 hours interval

KEGG category	Up-regulated Genes
**Peroxisome**	Phyx, Pex19, Paox, Crot, Mlycd
**mTOR signaling pathway**	Cab39l, Eif4b, Ulk1, Rps6kb2, Pgf
**Cytosolic DNA-sensing pathway**	Nfkbia, Mavs, Ikbke, Polr2e
**Sphingolipid metabolism**	Sphk1, Galc, Ppap2a
**SNARE interactions in vesicular transport**	Vamp1, Vamp4, Stx8

KEGG category	Down-regulated Genes
**MAPK signaling pathway**	Dusp6, Bndf, Hspa8, Ppp3ca, Prkca, Gadd45a, Mapkapk2, Pak1, Mecom, Dusp16, Rapgef2, Ngf, Rps6ka5, Rras2, Max, Stk3
**Pathways in cancer**	Cdk6, Pik3ca, Prkca, Stat5b, Arnt, Igf1r, E2f3, Skp2, Mecom, Pias1, Traf3, Rad51, Max, Bid, Ccdc6
**Focal adhesion**	Pik3ca, Prkca, Bcar1, Igf1r, Capn2, Mylk3, Pak1, Myl9, Vcl, Dock1, Myl12b, Ccnd2
**Regulation of actin cytoskeleton**	Pik3ca, Bcar1, Myh9, Mylk3, Pak1, Myl9, Vcl, Dock1, Rras2, Myl12b, Nckap, Msn
**Cell cycle**	Cdk6, Cdc25a, Gadd45a, E2f3, Skp2, Ccna, Mcm4, Cdc6, Anapc10, Ccnd2, Mad1l1
**Neurotrophin signaling pathway**	Pdk1, Pik3ca, Bdnf, Camk2d, Irs1, Mapkapk2, Ngf, Rps6ka5, Sh2b3
**RNA transport**	Eif4ebp1, Eif4e, Eif4a1, Nup93, Eif3a, Tmem48, Eif5, Pigz, Nup98
**ErbB signaling pathway**	Eif4ebp1, Pik3ca, Camk2d, Prkca, Stat5b, Hbegf, Pak1
**p53 signaling pathway**	Cdk6, Serpine1, Gadd45a, Ei24, Ccnd2, Bid
**Glioma**	Cdk6, Pik3ca, Camk2d, Prkca, Igf1r, E2f3
**Apoptosis**	Cflar, Pik3ca, Ppp3ca, Capn2, Ngf, Bid
**Progesterone-mediated oocyte maturation**	Pik3ca, Cdc25a, Igf1r, Ccna1, Anapc10, Mad1l1
**Ribosome biogenesis in eukaryotes**	Cirh1a, Mphosph10, Utp15, Heatr1, Xrn2, Gar1
**mTOR signaling pathway**	Eif4ebp1, Eif4e, Pik3ca, Prkaa1
**Circadian rhythm - mammal**	Npas2, Csnk1e, Csnk1d, Bhlhe40
**Steroid biosynthesis**	Hsd17b7, Nsdhl, Soat1

Table S12. Biological processes (GO) and respective enriched genes regulated by Ang II via AT2 receptor at 6 hours interval

Biological Process (GO)	Up-regulated Genes
positive regulation of cell proliferation	Sphk1, Il11, Casr, Erbb2, Stat5a, Ccnb1, Rogdi, Cd47, Myd88, Tbx18, Sox4, Cyba
protein transport	Dnajc14, Exoc3, Dnm2, Mcoln1, Nutf2, Tmed3, Duoxa1, Chmp1a, Gorasp1, Psen2, Sec16b
brain development	Stxbp3, Sphk1, Dmbx1, Tmem57, Bbc3, Fzd1, Tulp3, Cdk5rap3, Sharpin, Synj2
regulation of cell proliferation	Sphk1, Ndrg2, Erbb2, Mitf, Nfkbia, Ifi30, Creb3, Myd88, Cyba
vesicle-mediated transport	Stxbp3, Vamp1, Sar1a, Vamp4, Chmp1a, Stx8, Sec16b
inflammatory response	Sphk1, Lipa, Cyp4f5, Map2k3, Lias, Cyba
cell surface receptor linked signaling pathway	Erbb2, Fcer1g, Cd47, Myd88, Itga10, Plxnb1
I-kappaB kinase/ NF-kappaB cascade	Nfkbia, Ltbr, Myd88, Mavs, Ikbke, Sharpin
chromatin modification	Apbb1, Suv420h2, Smarcal1, Smarcd2
innate immune response	Myd88, Sp110, Mavs, Cyba
response to stress / oxygen levels	Ccnb1, Ndrg1, Bbc3, Asl

Biological Process (GO)	Down-regulated Genes
protein phosphorylation	Nek2, Cdk6, Pdk1, Pik3ca, Cdk12, Prkca, Igf1r, Mapkpk2, Mylk3, Pak1, Lmtk2, Lats2, Mast4, Pdik1l, Rps6ka5, MSt4, Nek7, Vrk2, Riok3, Mdfic, Epha2, Bmx, Gsk3a, Csnk1e, Csnk1d, Stk3, Prkaa1, Hipk2, Plk2
signal transduction	Stat5b, Il1rl1, Ahr, Rtkn, Ngf, Asap1, Traf3, Siva1, Syde1, Rras2, Stard13, Gsk3a, Pdpn, Ramp2, Arhgap17, Stk3, Arhgap10, Akap12, Gnb5, Plk2, Hmga2, Bpm7
apoptosis	SIah2, Hip1, Ahr, Nlrp3, Rtkn, Ngf, Fam82a, Nlrp1a, Bag4, Aen, Siva1, Pea15a, Epha2, Dap, Bid, Stk3
protein transport	Stxb1, Myh9, Abra, Nup93, Lmtk2, Ipo9, RUfy1, Tmem48, Rab6b, Pex14, Timm10, Vps45, Arf2, Nup98, Sec31a
negative regulation of apoptosis	Cflar, Pik3ca, Bdnf, Stat5b, Igf1r, Skp2, Btg2, Rtkn, Ngf, Ndufaf4, Siva1, Pea15a, Hipk3, Rtn4, Cat, Serpine1, Accn1, Cd44, Nes, Fignl1, Btg2, Ung, Tfap4, Wfs1, Bmp7
response to organic cyclic compound	Dusp6, Bdnf, Eno2, Prkca, Cd44, Ahr, Homer1, Btg2, Ngf, Atf1, Ctgf, Ccdnd2
regulation of cell proliferation	Cdk6, Serpine1, Prkca, Stat5b, Hbgef, Plcd3, Skp2, Mecom, Atp2b1, Mfn2, Bid, Rbpj
cell cycle	Cdk6, Siah2, Cdc25a, Ppp6c, Ahr, Spc25, Ccdc99, Thap1, Specc1l, Pdpn, Rgs2

**response to estrogen stimulus**	Eno2, Serpine1, Prkca, Stat5b, Ahr, Rbbp5, Ramp2, Ctgf, Ccnd2, Bid, Bmp7
**DNA replication**	Rfc2, Cdc25a, Kctd13, Mcm4, Clspn, Pole, Blm, Cdc6, Ctgf, Nup98
**regulation of apoptosis**	Cflar, Hip1, Mllt11, Deptor, Mst4, Traf3, Pea15a, Bid, Wfs1
**nervous system development**	Bdnf, Hbgef, Nes, Kat2a, Fnbp1l, Epha2, Slc7a5, Pdpn, Bhlhe40, Rtn4
**response to stress**	Bdnf, Cat, Hspa8, Serpine1, ppp3ca, Ahr, Homer1, Rps6ka5, Pdpn, Ctgf
**cell division**	Cdc5a, Ccna1, Spc25, Ccdc99, Top2a, Cdc6, Specc1l, Ccnd2
